# Near Field Models of Spatially-Fed Planar Arrays and Their Application to Multi-Frequency Direct Layout Optimization for mm-Wave 5G New Radio Indoor Network Coverage

**DOI:** 10.3390/s22228925

**Published:** 2022-11-18

**Authors:** Daniel R. Prado

**Affiliations:** Group of Signal Theory and Communications, Department of Electrical Engineering, Universidad de Oviedo, 33203 Gijón, Spain; drprado@uniovi.es

**Keywords:** spatially fed array, reflectarray antenna, transmitarray, near field, radiation equations, radiated field synthesis, optimization, electromagnetic field shaping, generalized intersection approach

## Abstract

Two near field models for the analysis of spatially fed planar array antennas are presented, compared and applied to a multi-frequency wideband direct layout optimization for mm-Wave 5G new radio (NR) indoor network coverage. One model is based on the direct application of the radiation equations directly derived from the A and F vector potentials. The second model is based on the superposition of far field contributions of all array elements, which are modelled as rectangular apertures with constant field. Despite the different assumptions made to develop both models, the degree of agreement between them in the computation of the radiated near field is very high. The relative error between the models is equal or lower than 3.2% at a plane 13λ from the array, and it decreases as the near field is computed further away from the array. Then, the faster model is employed in a general direct layout optimization procedure to shape the electromagnetic near field for application in an indoor femtocell to provide coverage with constant power in a private office. Results show that a magnitude ripple better than 1.5 dB can be achieved in an enlarged coverage area covering the whole n257 band of the 5G NR, corresponding to a 10.7% relative bandwidth.

## 1. Introduction

Wireless communications within the 3rd Generation Partnership Project (3GPP) 5G New Radio (NR) paradigm have introduced new concepts and technologies, including ultra-dense networking, massive machine communications, all-spectrum access, full-duplexing, and massive MIMO [1,2], for applications in the industry 4.0 [3], smart cities [4], Internet of Things (IoT) [5], precision agriculture [6], and self-driving cars [7], among others [8]. These applications require higher data rates, higher channel capacity, and lower latency than those achieved with previous generations of mobile networks [9]. To that end, the 3GPP 5G NR technical specification [10] identifies two frequency ranges (FR) for the implementation of the 5G networks, namely, FR1 (0.41–7.125 GHz) and FR2 (24.25–71 GHz), each of them divided into several bands. From the point of view of frequency allocation, the FR1 spectrum is already very crowded, by now providing services that range from cellular and satellite communications, to wireless local area networks [9]. Thus, the mm-wave ranges provided by the FR2, with their readily available wide spectrum, have become increasingly interesting for the deployment of 5G NR networks. In particular, there is special interest in the three lower bands in FR2, n257 (26.5–29.5 GHz), n258 (24.25–27.5 GHz), and n261 (27.5–28.35 GHz) since they have been released by the most countries, including the United States, the European Union, China, Japan, and South Korea [9]. However, the use of frequencies in the FR2 presents some challenges, such as path loss due to higher losses and shadowing due to blockage by physical objects that act as barriers for the propagating wave [11]. To overcome these issues, the use of femtocells with increased line of sight connectivity [12,13] has been proposed as a means of enhancing the cellular coverage while offloading the macro cell traffic in places with higher density.

Spatially fed arrays have been proposed within the intelligent reflective surface (IRS) paradigm [14,15,16], specifically reflectarray antennas for both indoor [17] and outdoor scenarios [18]. Transmitarrays have also been proposed as a solution to provide coverage in indoor femtocells [19,20]. In order to improve the coverage in indoor scenarios for transfer of information, power, or both simultaneously, near field beam-shaping techniques for the enlargement of coverage areas have been proposed. For instance, in [17] a phase-only synthesis (POS) based on the generalized intersection approach [21] was carried out to design two reflectarray antennas, one providing coverage in an outdoor scenario by considering a squared-cosecant beam in elevation and sectorial beam in azimuth, while the other reflectarray provides coverage in an indoor scenario. In [19], a novel near field POS algorithm was developed in which the far field resulting from the truncated near field according to specifications is computed as an intermediate step before recovering the field at the aperture by means of the fast Fourier transform (FFT). It was employed to design a 3D-printed dielectric transmitarray antenna that provides a very narrow coverage area. This algorithm was later extended [22] to include simultaneous near and far field constraints. Another near field POS is carried out in [20] to enlarge the coverage area provided by a multi-fed transmitarray antenna.

The techniques employed in the previous works are very efficient for the near field beam-shaping of spatially fed arrays at a single frequency. However, they present some shortcomings. One common characteristic of the previous works is that they perform a POS, where a phase-shift for each array element is obtained at a single frequency such that the array radiates the desired near field. From those phases, the chosen unit cell is tuned in such a way that it produces the required phase-shift [23]. One obvious limitation of such an approach is the narrow bandwidth nature of the obtained arrays. Indeed, since the POS and layout design only consider a single frequency, the in-band behaviour of the array rapidly deteriorates [24]. Moreover, due to the intermediate step of computing the far field in the near field synthesis of [19,22], that algorithm is restricted to a single near field plane that is parallel to the array aperture.

In order to overcome those limitations, this work proposes a general near field beam-shaping technique for the wideband optimization of spatially fed arrays. Specifically, the new analysis technique allows computation of the near field in a volume with an arbitrary local coordinate system rotated by three angles with regard to the array, as well as to perform a wideband direct layout optimization by taking into account the full electromagnetic response of the unit cell at several frequencies as provided by the use of a full-wave electromagnetic tool assuming local periodicity directly in the optimization loop. To that end, first two near field models for spatially fed arrays are presented. One model is based on the radiation equations directly derived from the A and F vector potentials [25]. The volumetric integrals are particularized to planar arrays discretized into a number of constituent elements. The resulting surface integrals are evaluated by means of the mid point quadrature, evaluating their convergence for different discretization grids. The second model is based on the superposition of the far fields radiated by each array element, which are modelled as small rectangular apertures with constant field on them. The far field is obtained with Love’s principle of equivalence for aperture antenna [26]. In addition, a formulation is given to have the possibility of computing the near field in an arbitrary local coordinate system which is rotated and translated with regard to the array local coordinate system, which might be useful depending on the application. Both near field models are then compared by considering the near field radiated by a transmitarray antenna, showing a high degree of agreement between the two models. Then, the faster model is applied to a novel technique for the wideband near field beam-shaping based on the generalized intersection approach (GIA) [21]. This technique performs a direct layout optimization employing a method of moments based on local periodicity (MoM-LP) directly in the optimization loop to account for the real electromagnetic response of the unit cell at several frequencies of interest, including mutual coupling between the array elements. In this way, a general near field multi-frequency optimization algorithm is obtained. As an example of application, it is applied to an indoor scenario in which it is desired to provide coverage in a private office with a 5G NR femtocell working in the n257 band, which comprises the frequency range 26.5 –29.5 GHz, corresponding to a 10.7% relative bandwidth. The optimization procedure with the GIA is divided into several stages to facilitate convergence towards a suitable solution. The final optimized array presents a maximum magnitude ripple in the enlarged coverage area of 1.5 dB over the whole n257 bandwidth, showing the suitability of the proposed methodology for the wideband near field beam-shaping of spatially fed array antennas.

## 2. Statement of the Problem

**Figure 1:**
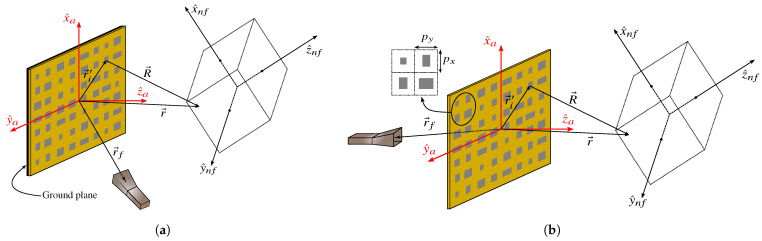
Sketch of the (**a**) reflectarray and (**b**) transmitarray scenarios. The goal is to obtain the near field radiated by the arrays in a volume in front of the antenna. The analysis of the arrays is carried out in the array coordinate system (ACS) defined by $\left({\hat{x}}_{a},{\hat{y}}_{a},{\hat{z}}_{a}\right)$, while the radiated field will be obtained in the near field coordinate system (NFCS) defined by $\left({\hat{x}}_{n\;f},{\hat{y}}_{n\;f},{\hat{z}}_{n\;f}\right)$. The transformation between ACS and NFCS may be consulted in Appendix A.

Let us consider a spatially fed planar array, namely a reflectarray or transmitarray, comprised of *N* elements with a regular periodicity of $p_{x}\times p_{y}$. The goal is to calculate the near field radiated by the array in a volume discretized in *M* points in front of the antenna and to shape the near field such that it complies with certain specifications for a given application. To that end, Figure 1 shows a sketch of the near field model for the two considered arrays. From the point of view of the analysis of the array, both spatially fed arrays are analysed in the same way, considering their own local coordinate system (ACS, array coordinate system) defined by $\left({\hat{x}}_{a},{\hat{y}}_{a},{\hat{z}}_{a}\right)$. The location (i.e., centre) of the *i*-th array element is given by ${\vec{r}}_{i}\;{\;}^{\prime }=\left(x_{i}^{\prime },y_{i}^{\prime },z_{i}^{\prime }=0\right)$, which is a point in the surface of the planar array, and $\vec{r}=\left(x,y,z\right)$ is a point in space in front of the array, i.e., with z>0. Both ${\vec{r}}_{i}\;{\;}^{\prime }$ and $\vec{r}$ are defined in the ACS, and $\vec{R}=\vec{r}-{\vec{r}}_{i}\;{\;}^{\prime }$.

On the other hand, the radiated field is referred to a rotated—and translated—coordinate system (NFCS, near field coordinate system) noted as $\left({\hat{x}}_{n\;f},{\hat{y}}_{n\;f},{\hat{z}}_{n\;f}\right)$. The relation between the ACS and NFCS is given by matrix *T*, which is derived in Appendix A. Since the analysis of the array is carried out in the ACS, the calculated radiated field will have to be transformed so that it is referenced to the NFCS.

In the following section, two near field models will be developed. One is based on the exact radiated field equations, while the other is based on the superposition of the far field radiated by each array element. In both cases, the radiation element is modelled as a small rectangular aperture with constant field on it.

## 3. Near Field Models of Reflectarray and Transmitarray Antennas

### 3.1. Field at the Aperture

Before presenting the two near field models, the field at the aperture needs to be calculated, since it is used as the source from which the radiated field is obtained. First, a feed whose phase center is placed at ${\vec{r}}_{f}=\left(x_{f},y_{f},z_{f}\right)$ in the ACS generates a tangential incident field on the surface of the array, which can be written for the *i*-th element as:

(1) $${\vec{E}}_{\mathrm{inc}}\left({\vec{r}}_{i}\;{\;}^{\prime }\right)=E_{\mathrm{inc},x}\left({\vec{r}}_{i}\;{\;}^{\prime }\right){\hat{x}}_{a}+E_{\mathrm{inc},y}\left({\vec{r}}_{i}\;{\;}^{\prime }\right){\hat{y}}_{a},$$

with i=1,…,N. In order to alleviate the notation, the dependence on ${\vec{r}}_{i}\;{\;}^{\prime }$ of the field components will be dropped from here on. From the incident field, the field at the aperture may be calculated as:

(2) $${\vec{E}}_{\mathrm{ap}}\left({\vec{r}}_{i}\;{\;}^{\prime }\right)=M_{i}{\vec{E}}_{\mathrm{inc}}\left({\vec{r}}_{i}\;{\;}^{\prime }\right)$$

where $M_{i}$ is a 2×2 matrix of complex numbers which depends on the considered array. For reflectarrays, $M_{i}$ is the matrix of reflection coefficients:

(3) $$M_{i}=\left(\begin{array}{cc} \rho_{xx,i} & \rho_{xy,i}\\ \rho_{yx,i} & \rho_{yy,i} \end{array}\right),$$

and the field at the aperture ${\vec{E}}_{\mathrm{ap}}$ would correspond to the reflected field. In the case of transmitarrays, $M_{i}$ is the matrix of transmission coefficients:

(4) $$M_{i}=\left(\begin{array}{cc} \tau_{xx,i} & \tau_{xy,i}\\ \tau_{yx,i} & \tau_{yy,i} \end{array}\right),$$

and the field at the aperture would correspond to the transmitted field. For both cases, $M_{i}$ is computed using a full-wave analysis tool assuming local periodicity. In addition, $M_{i}$ depends on the working frequency, unit cell topology, periodicity, substrate characteristics, and angles of incidence of the plane wave employed in the analysis [27].

Once the tangential electric field at the aperture has been obtained with (2), the tangential magnetic field may be calculated with:

(5) $${\vec{H}}_{\mathrm{ap}}\left({\vec{r}}_{i}\;{\;}^{\prime }\right)=\frac{\vec{k}\times {\vec{E}}_{\mathrm{ap}}\left({\vec{r}}_{i}\;{\;}^{\prime }\right)}{\omega \mu_{0}},$$

where

(6) $$\vec{k}=k_{x}{\hat{x}}_{a}+k_{y}{\hat{y}}_{a}+k_{z}{\hat{z}}_{a}=-k_{0}\left(\sin\theta_{i}\cos\varphi_{i}{\hat{x}}_{a}+\sin\theta_{i}\sin\varphi_{i}{\hat{y}}_{a}-\cos\theta_{i}{\hat{z}}_{a}\right),$$

ω=2πf, *f* is the frequency, $\mu_{0}$ is the free-space permeability, $k_{0}$ is the free-space wavenumber, and $\left(\theta_{i},\varphi_{i}\right)$ is the angle of incidence of the plane wave for the *i*-th array element (see Figure 1). The $\hat{z}$ component of ${\vec{E}}_{\mathrm{ap}}$, necessary to solve (5), is obtained by solving the plane wave equation $\vec{k}\cdot {\vec{E}}_{\mathrm{ap}}=0$. Then, the components of the reflected tangential magnetic field are:

(7a) $$\begin{array}{cc} H_{\mathrm{ap},x} & =-K_{1}E_{\mathrm{ap},x}-K_{2}E_{\mathrm{ap},y}, \end{array}$$

(7b) Hap,y=−K3Eap,x+K1Eap,y,

with:

(8a) $$\begin{array}{cc} K_{1} & =\frac{k_{x}k_{y}}{\omega \mu_{0}k_{z}}, \end{array}$$

(8b) $$\begin{array}{cc} K_{2} & =\frac{k_{y}^{2}+k_{z}^{2}}{\omega \mu_{0}k_{z}}, \end{array}$$

(8c) $$\begin{array}{cc} K_{3} & =\frac{k_{x}^{2}+k_{z}^{2}}{\omega \mu_{0}k_{z}}. \end{array}$$

In this way, the tangential field at the aperture is fully characterized by:

(9a) $$\begin{array}{cc} {\vec{E}}_{\mathrm{ap}}\left({\vec{r}}_{i}\;{\;}^{\prime }\right) & =E_{\mathrm{ap},x}{\hat{x}}_{a}+E_{\mathrm{ap},y}{\hat{y}}_{a}, \end{array}$$

(9b) $$\begin{array}{cc} {\vec{H}}_{\mathrm{ap}}\left({\vec{r}}_{i}\;{\;}^{\prime }\right) & =H_{\mathrm{ap},x}{\hat{x}}_{a}+H_{\mathrm{ap},y}{\hat{y}}_{a}, \end{array}$$

where ${\vec{E}}_{\mathrm{ap}}\left({\vec{r}}_{i}\;{\;}^{\prime }\right)$ is obtained from (2) and ${\vec{H}}_{\mathrm{ap}}\left({\vec{r}}_{i}\;{\;}^{\prime }\right)$ from (5) and may be readily employed as a source for the calculation of the radiated field.

Sometimes, the electric ($\vec{J}$) and magnetic ($\vec{M}$) current densities are required. Since planar arrays are considered, the notation ${\vec{J}}_{s}$ and ${\vec{M}}_{s}$ is employed. They can be obtained from the tangential field at the aperture as follows:

(10a) $$\begin{array}{cc} {\vec{J}}_{s}\left({\vec{r}}_{i}\;{\;}^{\prime }\right) & =\hat{n}\times {\vec{H}}_{\mathrm{ap}}\left({\vec{r}}_{i}\;{\;}^{\prime }\right)=H_{\mathrm{ap},x}{\hat{y}}_{a}-H_{\mathrm{ap},y}{\hat{x}}_{a}, \end{array}$$

(10b) $$\begin{array}{cc} {\vec{M}}_{s}\left({\vec{r}}_{i}\;{\;}^{\prime }\right) & =-\hat{n}\times {\vec{E}}_{\mathrm{ap}}\left({\vec{r}}_{i}\;{\;}^{\prime }\right)=-E_{\mathrm{ap},x}{\hat{y}}_{a}+E_{\mathrm{ap},y}{\hat{x}}_{a}, \end{array}$$

where $\hat{n}={\hat{z}}_{a}$ is the unit vector normal to the surface of the planar array. Please notice that the surface current densities are also tangential vectors, and thus the longitudinal components are zero, i.e., $J_{z}=M_{z}=0$.

### 3.2. Near Field from the Radiation Equations

The first near field model is based on the radiation equations obtained as superposition of the contributions of the vector potentials $\vec{A}$ and $\vec{F}$ [25]:

(11a) $$\begin{array}{cc} {\vec{E}}_{\mathrm{NF}}\left(\vec{r}\right) & ={\vec{E}}_{A}\left(\vec{r}\right)+{\vec{E}}_{F}\left(\vec{r}\right), \end{array}$$

(11b) $$\begin{array}{cc} {\vec{H}}_{\mathrm{NF}}\left(\vec{r}\right) & ={\vec{H}}_{A}\left(\vec{r}\right)+{\vec{H}}_{F}\left(\vec{r}\right), \end{array}$$

where

(12a) $$\begin{array}{cc} {\vec{E}}_{A}\left(\vec{r}\right) & =E_{A_{x}}{\hat{x}}_{a}+E_{A_{y}}{\hat{y}}_{a}+E_{A_{z}}{\hat{z}}_{a} \end{array}$$

(12b) $$\begin{array}{cc} {\vec{E}}_{F}\left(\vec{r}\right) & =E_{F_{x}}{\hat{x}}_{a}+E_{F_{y}}{\hat{y}}_{a}+E_{F_{z}}{\hat{z}}_{a} \end{array}$$

(12c) $$\begin{array}{cc} {\vec{H}}_{A}\left(\vec{r}\right) & =H_{A_{x}}{\hat{x}}_{a}+H_{A_{y}}{\hat{y}}_{a}+H_{A_{z}}{\hat{z}}_{a} \end{array}$$

(12d) $$\begin{array}{cc} {\vec{H}}_{F}\left(\vec{r}\right) & =H_{F_{x}}{\hat{x}}_{a}+H_{F_{y}}{\hat{y}}_{a}+H_{F_{z}}{\hat{z}}_{a} \end{array}$$

In (12) and subsequent equations, the dependence of the field components on the observation point $\vec{r}$ has been dropped to alleviate the notation. There are a total of 12 field components in (11), for which an integral of field sources must be evaluated. An example of one such component is:

(13) $$E_{F_{z}}=-\frac{1}{4\pi }{\;\int \int \int \;}_{V}[\left(y-y^{\prime }\right)M_{x}-\left(x-x^{\prime }\right)M_{y}]\frac{1+j\beta R}{R^{3}}e^{-j\beta R}dx^{\prime }dy^{\prime }dz^{\prime },$$

where *R* is the distance from a source point in the volume *V* to the observation point, and β is the phase constant that in this case is equal to the free-space wavenumber $k_{0}$. The rest of the integrals may be consulted in [25] (pp. 285–286). Since the arrays are planar, (13) can be reduced to:

(14) $$E_{F_{z}}=-\frac{1}{4\pi }{\;\int \int \;}_{S}[\left(y-y^{\prime }\right)M_{x}-\left(x-x^{\prime }\right)M_{y}]\frac{1+j\beta R}{R^{3}}e^{-j\beta R}dx^{\prime }dy^{\prime },$$

where *S* is the surface of the array. The array is divided into *N* unit cells, where the elements of matrix $M_{i}$, and thus the field at the aperture and surface currents, are computed. Thus, we express the integral as a sum of the integral over each array element:

(15) $$E_{F_{z}}=-\frac{1}{4\pi }\sum_{i=1}^{N}{\;\int \int \;}_{S_{i}}[\left(y-y^{\prime }\right)M_{x_{i}}-\left(x-x^{\prime }\right)M_{y_{i}}]\frac{1+j\beta R_{i}}{R_{i}^{3}}e^{-j\beta R_{i}}dx^{\prime }dy^{\prime },$$

where $S_{i}$ is the surface of the *i*-th unit cell, and:

(16) $$R_{i}=\left|\vec{r}-{\vec{r}}_{i}\;{\;}^{\prime }\right|=\sqrt{{\left(x-x_{i}\;{\;}^{\prime }\right)}^{2}+{\left(y-y_{i}\;{\;}^{\prime }\right)}^{2}+z^{2}}.$$

To finally obtain $E_{F_{z}}$ in (15), the double integral may be evaluated by any quadrature method, for instance, the mid point quadrature. With this technique, the integral is approximated by a double sum:

(17) $$\begin{array}{cc} V_{i}={\;\int \int \;}_{S_{i}}[\left(y-y^{\prime }\right)M_{x_{i}}-\left(x-x^{\prime }\right)M_{y_{i}}]\frac{1+j\beta R_{i}}{R_{i}^{3}}e^{-j\beta R_{i}} & dx^{\prime }dy^{\prime }\approx \sum_{h=1}^{N_{x}}\sum_{k=1}^{N_{y}}[\left(y-y^{\prime }\right)M_{x_{i}}-\\ & \left(x-x^{\prime }\right)M_{y_{i}}]\frac{1+j\beta R_{ihk}}{R_{ihk}^{3}}e^{-j\beta R_{ihk}}\Delta x\Delta y, \end{array}$$

where surface $S_{i}$ is divided into $N_{x}N_{y}$ rectangles of length $\Delta_{x}=p_{x}/N_{x}$ and width $\Delta_{y}=p_{y}/N_{y}$, and:

(18) $$R_{ihk}=\sqrt{{\left(x-x_{ih}\right)}^{2}+{\left(y-y_{ki}\right)}^{2}+z^{2}},$$

with:

(19a) $$\begin{array}{cc} x_{ih} & =-p_{x}/2+\left(h-1/2\right)\Delta_{x}+x_{i}\;{\;}^{\prime }, \end{array}$$

(19b) $$\begin{array}{cc} y_{ik} & =-p_{y}/2+\left(k-1/2\right)\Delta_{y}+y_{i}\;{\;}^{\prime }. \end{array}$$

Thus, the function is evaluated at the center of each rectangle. By substituting (17) into (15), we finally obtain:

(20) $$E_{F_{z}}=-\frac{1}{4\pi }\sum_{i=1}^{N}\sum_{h=1}^{N_{x}}\sum_{k=1}^{N_{y}}[\left(y-y^{\prime }\right)M_{x_{i}}-\left(x-x^{\prime }\right)M_{y_{i}}]\frac{1+j\beta R_{ihk}}{R_{ihk}^{3}}e^{-j\beta R_{ihk}}\Delta x\Delta y.$$

The same process can be applied to the other 11 integrals in [25] (pp. 285–286), taking into account that the planar arrays are placed at the plane $z^{\prime }=0$ and that the longitudinal components of the surface currents are zero ($J_{z}=M_{z}=0$). For the sake of completeness, the final expressions for all the integrals particularized for spatially fed planar arrays are gathered next:

(21a) $$\begin{array}{cc} E_{A_{x}} & =\frac{-j\eta }{4\pi \beta }\sum_{i=1}^{N}\sum_{h=1}^{N_{x}}\sum_{k=1}^{N_{y}}\{G_{1}J_{x_{i}}+\left(x-x^{\prime }\right)G_{2}\left[\left(x-x^{\prime }\right)J_{x_{i}}+\left(y-y^{\prime }\right)J_{y_{i}}\right]\}e^{-j\beta R_{ihk}}\Delta x\Delta y, \end{array}$$

(21b) $$\begin{array}{cc} E_{A_{y}} & =\frac{-j\eta }{4\pi \beta }\sum_{i=1}^{N}\sum_{h=1}^{N_{x}}\sum_{k=1}^{N_{y}}\{G_{1}J_{y_{i}}+\left(y-y^{\prime }\right)G_{2}\left[\left(x-x^{\prime }\right)J_{x_{i}}+\left(y-y^{\prime }\right)J_{y_{i}}\right]\}e^{-j\beta R_{ihk}}\Delta x\Delta y, \end{array}$$

(21c) $$\begin{array}{cc} E_{A_{z}} & =\frac{-j\eta }{4\pi \beta }\sum_{i=1}^{N}\sum_{h=1}^{N_{x}}\sum_{k=1}^{N_{y}}zG_{2}\left[\left(x-x^{\prime }\right)J_{x_{i}}+\left(y-y^{\prime }\right)J_{y_{i}}\right]e^{-j\beta R_{ihk}}\Delta x\Delta y, \end{array}$$

(21d) $$\begin{array}{cc} E_{F_{x}} & =\frac{-1}{4\pi }\sum_{i=1}^{N}\sum_{h=1}^{N_{x}}\sum_{k=1}^{N_{y}}zM_{y_{i}}\frac{1+j\beta R_{ihk}}{R_{ihk}^{3}}e^{-j\beta R_{ihk}}\Delta x\Delta y, \end{array}$$

(21e) $$\begin{array}{cc} E_{F_{y}} & =\frac{-1}{4\pi }\sum_{i=1}^{N}\sum_{h=1}^{N_{x}}\sum_{k=1}^{N_{y}}-zM_{x_{i}}\frac{1+j\beta R_{ihk}}{R_{ihk}^{3}}e^{-j\beta R_{ihk}}\Delta x\Delta y, \end{array}$$

(21f) $$\begin{array}{cc} E_{F_{z}} & =\frac{-1}{4\pi }\sum_{i=1}^{N}\sum_{h=1}^{N_{x}}\sum_{k=1}^{N_{y}}[\left(y-y^{\prime }\right)M_{x_{i}}-\left(x-x^{\prime }\right)M_{y_{i}}]\frac{1+j\beta R_{ihk}}{R_{ihk}^{3}}e^{-j\beta R_{ihk}}\Delta x\Delta y, \end{array}$$

(21g) $$\begin{array}{cc} H_{A_{x}} & =\frac{1}{4\pi }\sum_{i=1}^{N}\sum_{h=1}^{N_{x}}\sum_{k=1}^{N_{y}}zJ_{y_{i}}\frac{1+j\beta R_{ihk}}{R_{ihk}^{3}}e^{-j\beta R_{ihk}}\Delta x\Delta y, \end{array}$$

(21h) $$\begin{array}{cc} H_{A_{y}} & =\frac{1}{4\pi }\sum_{i=1}^{N}\sum_{h=1}^{N_{x}}\sum_{k=1}^{N_{y}}-zJ_{x_{i}}\frac{1+j\beta R_{ihk}}{R_{ihk}^{3}}e^{-j\beta R_{ihk}}\Delta x\Delta y, \end{array}$$

(21i) $$\begin{array}{cc} H_{A_{z}} & =\frac{1}{4\pi }\sum_{i=1}^{N}\sum_{h=1}^{N_{x}}\sum_{k=1}^{N_{y}}[\left(y-y^{\prime }\right)J_{x_{i}}-\left(x-x^{\prime }\right)J_{y_{i}}]\frac{1+j\beta R_{ihk}}{R_{ihk}^{3}}e^{-j\beta R_{ihk}}\Delta x\Delta y, \end{array}$$

(21j) $$\begin{array}{cc} H_{F_{x}} & =\frac{-j}{4\pi \beta \eta }\sum_{i=1}^{N}\sum_{h=1}^{N_{x}}\sum_{k=1}^{N_{y}}\{G_{1}M_{x_{i}}+\left(x-x^{\prime }\right)G_{2}\left[\left(x-x^{\prime }\right)M_{x_{i}}+\left(y-y^{\prime }\right)M_{y_{i}}\right]\}e^{-j\beta R_{ihk}}\Delta x\Delta y, \end{array}$$

(21k) $$\begin{array}{cc} H_{F_{y}} & =\frac{-j}{4\pi \beta \eta }\sum_{i=1}^{N}\sum_{h=1}^{N_{x}}\sum_{k=1}^{N_{y}}\{G_{1}M_{y_{i}}+\left(y-y^{\prime }\right)G_{2}\left[\left(x-x^{\prime }\right)M_{x_{i}}+\left(y-y^{\prime }\right)M_{y_{i}}\right]\}e^{-j\beta R_{ihk}}\Delta x\Delta y, \end{array}$$

(21l) $$\begin{array}{cc} H_{F_{z}} & =\frac{-j}{4\pi \beta \eta }\sum_{i=1}^{N}\sum_{h=1}^{N_{x}}\sum_{k=1}^{N_{y}}zG_{2}\left[\left(x-x^{\prime }\right)M_{x_{i}}+\left(y-y^{\prime }\right)M_{y_{i}}\right]e^{-j\beta R_{ihk}}\Delta x\Delta y, \end{array}$$

where η is the free-space impedance, and:

(22a) $$\begin{array}{cc} G_{1} & =\frac{-1-j\beta R_{ihk}+\beta^{2}R_{ihk}^{2}}{R_{ihk}^{3}}, \end{array}$$

(22b) $$\begin{array}{cc} G_{2} & =\frac{3+3j\beta R_{ihk}-\beta^{2}R_{ihk}^{2}}{R_{ihk}^{5}}. \end{array}$$

### 3.3. Near Field as Superposition of Far Field Contributions

**Figure 2:**
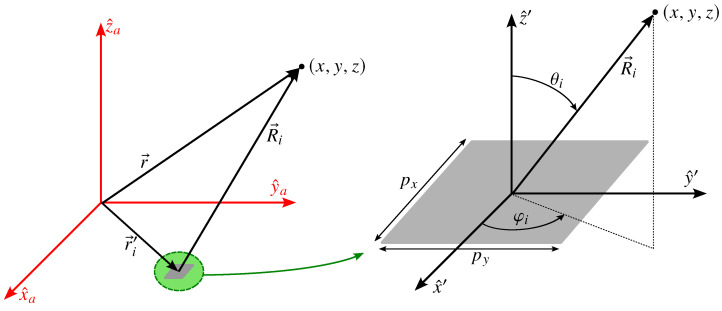
The definition of the angles $\theta_{i}$ and $\varphi_{i}$ for the computations of the far field radiated by an array element are defined in the element coordinate system $\left({\hat{x}}^{\prime },{\hat{y}}^{\prime },{\hat{z}}^{\prime }\right)$, which is the same as the ACS but translated by ${\vec{r}}_{i}\;{\;}^{\prime }$.

To develop this model, we have to consider each unit cell in the array as a unit of radiation and calculate its far field. In such a case, the total near field will be the superposition of the far fields radiated by each element:

(23a) $$\begin{array}{cc} {\vec{E}}_{\mathrm{NF}}\left(\vec{r}\right) & =\sum_{i=1}^{N}{\vec{E}}_{\mathrm{FF},i}\left(\vec{r}\right) \end{array}$$

(23b) $$\begin{array}{cc} {\vec{H}}_{\mathrm{NF}}\left(\vec{r}\right) & =\sum_{i=1}^{N}{\vec{H}}_{\mathrm{FF},i}\left(\vec{r}\right), \end{array}$$

where ${\vec{E}}_{\mathrm{FF},i}$ and ${\vec{H}}_{\mathrm{FF},i}$ are calculated as the far field radiated by an aperture [26]. The electric far field is:

(24) $${\vec{E}}_{\mathrm{FF},i}=E_{\theta ,i}\hat{\theta }+E_{\varphi ,i}\hat{\varphi },$$

and its components according to Love’s equivalent principle:

(25a) $$\begin{array}{cc} E_{\theta ,i} & =\frac{jk_{0}e^{-jk_{0}R_{i}}}{4\pi R_{i}}\left[P_{x,i}\cos\varphi_{i}+P_{y,i}\sin\varphi_{i}-\;\eta \cos\theta_{i}\left(Q_{x,i}\sin\varphi_{i}-Q_{y,i}\cos\varphi_{i}\right)\right], \end{array}$$

(25b) $$\begin{array}{cc} E_{\varphi ,i} & =-\frac{jk_{0}e^{-jk_{0}R_{i}}}{4\pi R_{i}}\left[\eta \left(Q_{x,i}\cos\varphi_{i}+Q_{y,i}\sin\varphi_{i}\right)+\;\cos\theta_{i}\left(P_{x,i}\sin\varphi_{i}-P_{y,i}\cos\varphi_{i}\right)\right], \end{array}$$

where the angles $\theta_{i}$ and $\varphi_{i}$ are defined in Figure 2.

*P* and *Q* are known as the spectrum functions, and they are the Fourier transform of the field at the aperture:

(26) $$\begin{array}{cc} P_{x/y,i} & ={\;\int \int \;}_{S_{i}}E_{\mathrm{ap},x/y,i}\left(x,y\right)\;e^{jk_{0}\left(ux+vy\right)}\;dx\;dy,\\ Q_{x/y,i} & ={\;\int \int \;}_{S_{i}}H_{\mathrm{ap},x/y,i}\left(x,y\right)\;e^{jk_{0}\left(ux+vy\right)}\;dx\;dy, \end{array}$$

where $u=\sin\theta_{i}\cos\varphi_{i}$ and $v=\sin\theta_{i}\sin\varphi_{i}$. Since the aperture is element *i* of the array, we consider a constant field, which comes out of the integral, yielding:

(27a) $$\begin{array}{cc} P_{x/y,i} & =E_{\mathrm{ap},x/y,i}{\;\int \int \;}_{S_{i}}e^{jk_{0}\left(ux+vy\right)}\;dx\;dy, \end{array}$$

(27b) $$\begin{array}{cc} Q_{x/y,i} & =H_{\mathrm{ap},x/y,i}{\;\int \int \;}_{S_{i}}e^{jk_{0}\left(ux+vy\right)}\;dx\;dy. \end{array}$$

The double integral in (27) can be solved analytically yielding [28]:

(28) $${\;\int \int \;}_{S_{i}}e^{jk_{0}\left(ux+vy\right)}\;dx\;dy=p_{x}p_{y}\mathrm{sinc}\left(\frac{k_{0}up_{x}}{2}\right)\mathrm{sinc}\left(\frac{k_{0}vp_{y}}{2}\right),$$

where sincx=sinx/x is the unnormalized sinc function.

Once the electric far field has been obtained, the magnetic far field may be obtained using the plane wave relation:

(29) $${\vec{H}}_{\mathrm{FF},i}=\frac{\hat{r}\times {\vec{E}}_{\mathrm{FF},i}}{\eta }=\frac{E_{\varphi ,i}\hat{\theta }+E_{\theta ,i}\hat{\varphi }}{\eta }.$$

The far field of an array element obtained with (24) and (29) is expressed in spherical coordinates, while we are interested in obtaining the near field in a Cartesian basis. Thus, to perform the superposition of (23), the far field of an array element must be expressed as:

(30a) $$\begin{array}{cc} {\vec{E}}_{\mathrm{FF},i} & =E_{\mathrm{FF},x,i}{\hat{x}}_{a}+E_{\mathrm{FF},y,i}{\hat{y}}_{a}+E_{\mathrm{FF},z,i}{\hat{z}}_{a}, \end{array}$$

(30b) $$\begin{array}{cc} {\vec{H}}_{\mathrm{FF},i} & =H_{\mathrm{FF},x,i}{\hat{x}}_{a}+H_{\mathrm{FF},y,i}{\hat{y}}_{a}+H_{\mathrm{FF},z,i}{\hat{z}}_{a}, \end{array}$$

where the Cartesian components are found with:

(31) $$\left(\begin{array}{c} E_{\mathrm{FF},x,i}\\ E_{\mathrm{FF},y,i}\\ E_{\mathrm{FF},z,i} \end{array}\right)=\left(\begin{array}{cc} \cos\theta_{i}\cos\varphi_{i} & -\sin\varphi_{i}\\ \cos\theta_{i}\sin\varphi_{i} & \cos\varphi_{i}\\ -\sin\theta & 0 \end{array}\right)\left(\begin{array}{c} E_{\theta }\\ E_{\varphi } \end{array}\right).$$

The same procedure is applied to the ${\vec{H}}_{\mathrm{FF}}$ field.

### 3.4. Change of Coordinates

The near field obtained with either (11) or (23) is referenced to the ACS. Moreover, to compute the near field following the equations derived in the previous subsections, the point in space $\vec{r}=\left(x,y,z\right)$ must also be expressed in the ACS. However, the goal is to obtain the near field in the NFCS, since the array may be rotated with regard to the desired local coordinate system (for instance, if the reflectarray is placed on a wall, and it is desired to provide indoor coverage on a table).

In general, the coordinates of the points where the near field is to be computed will be initially given in the NFCS. Thus, in order to compute the near field with (11) and (23), these coordinates need to be transformed from the NFCS to the ACS as detailed in Appendix A. Then, after the near field is computed in the ACS, it needs to be transformed to the NFCS as follows:

(32a) $$\begin{array}{cc} {\left.{\vec{E}}_{\mathrm{NF}}\left(\vec{r}\right)\right|}_{\mathrm{NFCS}} & =T\;{\left.{\vec{E}}_{\mathrm{NF}}\left(\vec{r}\right)\right|}_{\mathrm{ACS}}, \end{array}$$

(32b) $$\begin{array}{cc} {\left.{\vec{H}}_{\mathrm{NF}}\left(\vec{r}\right)\right|}_{\mathrm{NFCS}} & =T\;{\left.{\vec{H}}_{\mathrm{NF}}\left(\vec{r}\right)\right|}_{\mathrm{ACS}}, \end{array}$$

where *T* is defined in Appendix A, and ${\left.{\vec{E}}_{\mathrm{NF}}\left(\vec{r}\right)\right|}_{\mathrm{ACS}}$ and ${\left.{\vec{H}}_{\mathrm{NF}}\left(\vec{r}\right)\right|}_{\mathrm{ACS}}$ are the same as in (11) and (23).

### 3.5. Comparison of the Near Field Models

To compare the near field predicted by both models, let us consider a center-fed square planar transmitarray comprised of 60×60 elements in a regular grid of periodicity 3.84 mm^2^. The feed is modelled as a $\cos^{q}\theta$ function [29] with q=22 and placed at ${\vec{r}}_{f}=\left(0,0,-180\right)\;\mathrm{mm}$ in the ACS (see Figure 1). The working frequency is 39 GHz. For the analysis, the phases of the direct transmission coefficients in (4) are set to:

(33) $$∠\tau_{xx}=∠\tau_{yy}=k_{0}\left(d_{i}-\left(x^{\prime }\cos\varphi_{0}+y^{\prime }\sin\varphi_{0}\right)\sin\theta_{0}\right),$$

where $d_{i}$ is the distance from the feed phase center to the *i*-th array element, and $\left(\theta_{0},\varphi_{0}\right)$ is the pointing direction of the far field pencil beam defined by the phases of $\tau_{xx}$ and $\tau_{yy}$. For the present example, $\left(\theta_{0},\varphi_{0}\right)$ is set to $\left(0^{{}^{\circ}},0^{{}^{\circ}}\right)$.

The radiated near field will be calculated with the two models in planes parallel to the array with *z* constant and values z=0.1m, 1m. Thus, the matrix of change of coordinates from the ACS to the NFCS is the identity. In addition, in the case of the model based on the radiation equations, it will be evaluated for several values of $N_{x}$ and $N_{y}$, specifically for $N_{x}=N_{y}=1,3,5$ to assess the convergence of the integrals. Finally, each plane is divided into a grid of 150×150 points (22,500 in total) where the near field is computed.

**Figure 3:**
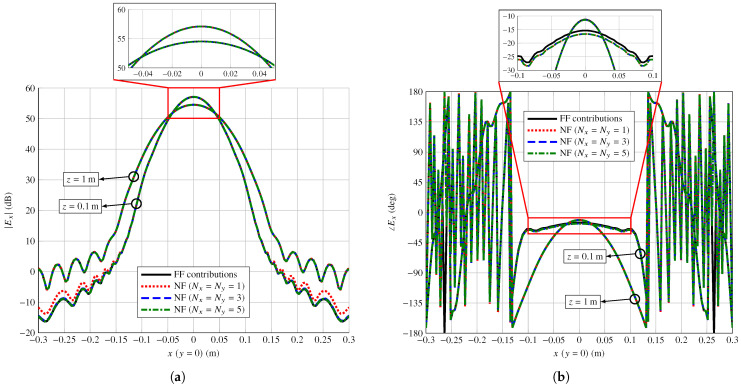
Comparison of the near field radiated by a center-fed transmitarray in (**a**) magnitude and (**b**) phase at the different *z* planes as calculated by the near field model based on the superposition of far field (FF) contributions of each array element, and the near field (NF) model based on the radiation equations for different values of $N_{x}$ and $N_{y}$ to assess the convergence in the numerical evaluation of the double integrals. Near field is plotted in the NFCS.

Figure 3 shows the main cuts in *x* for y=0 for the near field component $E_{x}$ in magnitude and phase for both *z* planes. The agreement between both models is in general very good. In the case of the magnitude, the model based on the radiation equations with $N_{x}=N_{y}=1$ presents some discrepancies in the plane z=0.1m with the rest of the models, albeit for very low values, 60 dB lower than the peak magnitude. In the case of the phase, the model based on the superposition of the far field radiated by each array element presents a small discrepancy in the plane z=0.1m. In this case, the shape of the phase is the same as in the other models, but it has a shift of approximately 1.3 deg. Both discrepancies disappear when the field is calculated further away from the array.

**Figure 4:**
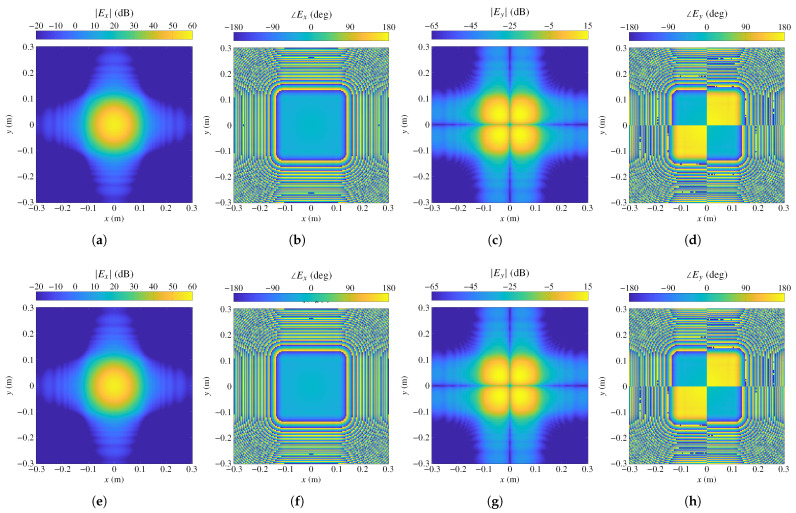
Comparison of the near field radiated by a center-fed transmitarray in magnitude and phase for the plane z=0.1m generated by the model based on the superposition of far field contributions of each array element (**top** row), and the near field model based on the radiation equations for $N_{x}=N_{y}=5$ (**bottom** row). The relative error between both simulations taking the bottom row as reference is 2.2% for both components of the field. Near field is plotted in the NFCS.

Figure 4 shows the magnitude and phase of the field components $E_{x}$ and $E_{y}$ as calculated by the models based on far field superposition (top row) and the radiation equations with $N_{x}=N_{y}=5$ (bottom row) for the plane z=0.1m. As can be seen, visually the results are practically the same.

A better way of assessing the similarities between both models is to compute the relative error in the calculated near fields. To that end, the following expression is employed:

(34) $$\mathrm{RE}=100\frac{∥E_{x|y|z}-{\tilde{E}}_{x|y|z}∥}{∥E_{x|y|z}∥},$$

where ∥·∥ is the Euclidean norm; *E* is one component of the near field computed with the model based on the radiation equations with $N_{x}=N_{y}=5$, which is considered as reference to compute the relative error in percentage; and $\tilde{E}$ is the same component of the near field computed with any other model. Table 1 shows the relative error for both models. It shows the relative errors for the three components of the near field for both planes. In the case of the model based on the radiation equations, even a value of $N_{x}=N_{y}=1$ shows a sufficiently low error. As the near field is computed further away from the array, the error decreases. The use of $N_{x}=N_{y}=1$ means that the double integrals in (15) are just evaluated at the center of the array element, i.e., considering only one sample, effectively reducing the triple summation in (21) to a single sum, considerably accelerating computations without barely affecting accuracy in the prediction of the near field. In the case of the near field model based on the superposition of the far fields radiated by each array element, the relative error is slightly larger, and it also decreases as the near field is computed further away from the array. However, even for a plane at z=0.1m, which is 13λ at the working frequency, the error is very low.

In light of these results, it is clear that both models offer practically the same results in the computation of the near field for spatially fed planar arrays. However, from a computational point of view, the model based on the superposition of far fields is more interesting. Indeed, as shown in Table 2, this model is at least one order of magnitude faster than the model based on the radiation equations. This is particularly important when employing the model in optimization procedures to shape the near field, since the analysis routine where the near field is computed is invoked many times. Then, the model based on the radiation equations could be used, if desired, to assess the validity of the results that were obtained employing the approximate model based on superposition of the far fields radiated by the array elements.

## 4. Wideband near Field Shaping with Application to Indoor Femtocell Coverage

### 4.1. Scenario Definition and Antenna Specifications

**Figure 5:**
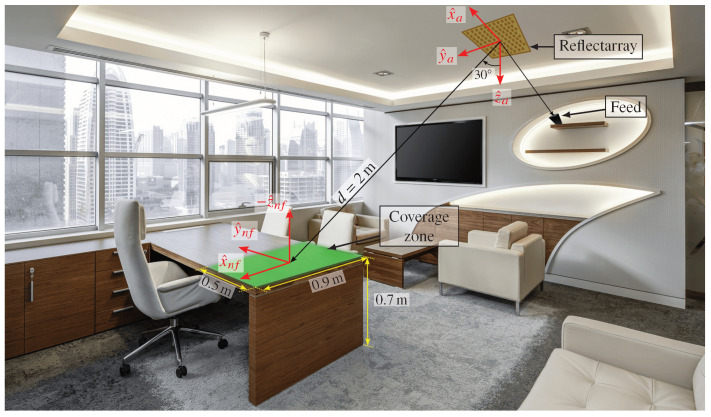
Proposed scenario for indoor femtocell coverage. A reflectarray provides a coverage on top of an office desktop with a maximum desired magnitude ripple of 2 dB. Picture adapted from [31].

As an example of application of the developed models for near field beam-shaping, let us consider the scenario depicted in Figure 5, where the goal is to provide coverage on top of an office desktop. The coverage area is a rectangle of dimensions $0.9\times 0.5\;m^{2}$ on the right side of the table. The reflectarray that provides the coverage is placed on the ceiling, at a distance of 2 m from the center of the coverage area, while the feed is placed on a shelf to illuminate the reflectarray. The goal is to achieve a maximum magnitude ripple in the coverage area of 2 dB in the whole 5G NR n257 band (26.5–29.5 GHz) in dual-linear polarization.

The considered reflectarray is square and comprised of N=1936 elements in a regular grid of 44×44. The periodicity is 5.35 mm in both dimensions, which is approximately half a wavelength at the central frequency of operation, 28 GHz. The phase center of the feed is placed at ${\vec{r}}_{f}=\left(-0.16,0,0.24\right)\;m$ with regard to the reflectarray center and generates an average edge illumination taper between −14.7 dB and −16.5 dB in the range 26.5–29.5 GHz.

### 4.2. Unit Cell Characterization

**Figure 6:**
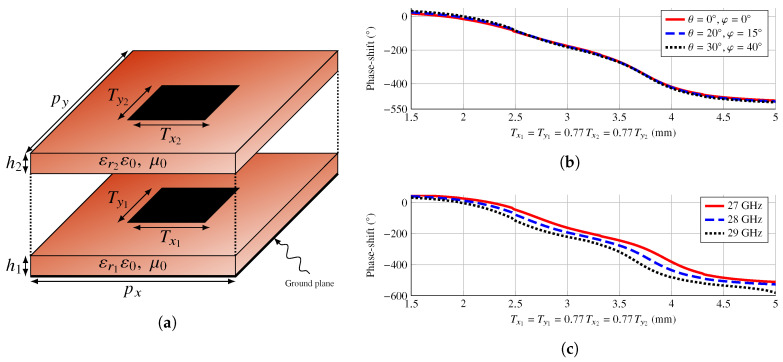
(**a**) Sketch of the employed unit cell, consisting of two stacked rectangular patches backed by a ground plane. The periodicity is $p_{x}=p_{y}=5.35\;\mathrm{mm}$, and both layers use the same substrate from Rogers Co., DiClad 870 with thickness of 30 mil; (**b**) phase response of the unit cell for normal and oblique incidence, showing that the unit cell response is stable with the angle of incidence; (**c**) phase response of the unit cell for three different frequencies for oblique incidence with $\left(\theta =40^{{}^{\circ}},\varphi =30^{{}^{\circ}}\right)$.

In order to be able to successfully perform a wideband direct layout optimization, the chosen unit cell should be able to provide a large enough phase-shift range as well as enough degrees of freedom for the optimization. In this regard, a unit cell comprised of only one rectangular patch would not provide enough phase-shift and degrees of freedom for its use in a wideband optimization procedure [32]. However, by stacking two rectangular patches, the range of the phase-shift can be increased. Thus, the chosen unit cell consists of two stacked rectangular patches backed by a ground plane as shown in Figure 6a. Both layers employ the same substrate, the Rogers DiClad 870 with relative permittivity $\varepsilon_{r}=2.33$, loss tangent tanδ=0.005, and thickness h=30mil. A CuClad 6250 bonding film between the two layers with the same electrical characteristics as the DiClad 870 is considered in the simulations. The substrate has been chosen such that a large enough phase-shift range is achieved.

For the initial layout design after the POS (see Section 4.3), a fixed relative size of the stacked patches is kept, with $T_{x_{1}}=0.77T_{x_{2}}$ and $T_{y_{1}}=0.77T_{y_{2}}$. The scaling factors were found after performing a parametric study to obtain a smooth variation of the phase-shift for $T_{x_{1}}=T_{y_{1}}$ at several frequencies and angles of incidence. Figure 6b shows the phase response of the unit cell at 28 GHz for normal and oblique incidence. The total phase-shift provided by the cell with the selected substrate is around 500°, and it can be seen that it is stable with the angle of incidence. Conversely, Figure 6c shows the unit cell phase response at three different frequencies for oblique incidence with $\left(\theta =40^{{}^{\circ}},\varphi =30^{{}^{\circ}}\right)$. From these curves, it can be seen that the unit cell also provides around 500° of phase-shift at several frequencies, making it suitable for a multi-frequency optimization.

### 4.3. Phase-Only Synthesis at 28 GHz

**Figure 7:**
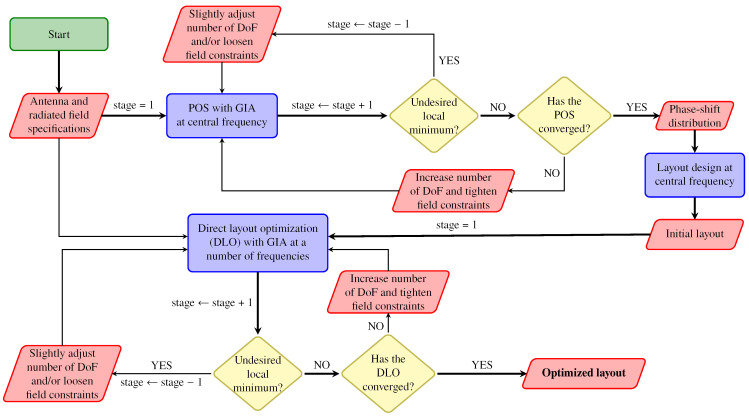
Proposed wideband optimization strategy for spatially fed arrays using the generalized intersection approach (GIA). First, a phase-only synthesis (POS) at central frequency is carried out in order to obtain a phase-shift distribution from which the initial layout is obtained. Then, the layout is used in a direct layout optimization (DLO) at multiple frequencies until specifications are met.

Figure 7 shows the proposed wideband near field optimization strategy for spatially fed array antennas. It is divided into several stages in order to facilitate convergence towards a suitable solution. After defining the antenna and near field specifications, a phase-only synthesis is carried out at central frequency. Afterwards, the geometrical features of the unit cell are tuned so each element produces the desired phase-shift obtained from the POS. After this point, the near field radiated by the antenna closely complies with the specifications at central frequency. However, due to the narrow bandwidth nature of planar spatially fed arrays, it does not comply at other frequencies. Thus, this initial layout is used in a multi-stage direct layout optimization (DLO) at several frequencies, finally obtaining an optimized wideband layout. To obtain an array working in dual-linear polarization, the POS is carried out independently for both polarizations, while the DLO is performed considering specifications in both polarizations simultaneously.

For the POS, the generalized intersection approach (GIA) [33] will be employed. The POS assumes an ideal phase-shifter simplification in matrix $M_{i}$, in which there are no losses ($|\rho_{xx,i}\left|=\right|\rho_{yy,i}|=1$ for reflectarrays, or $|\tau_{xx,i}\left|=\right|\tau_{yy,i}|=1$ for transmitarrays) and no cross-polarization ($\rho_{xy,i}=\rho_{yx,i}=0$ for reflectarrays, or $\tau_{xy,i}=\tau_{yx,i}=0$ for transmitarrays). In this way, only the phases of the direct coefficients are considered as optimization variables. Once the POS has converged to a near field that complies (or it is close to compliance) with the imposed specifications at central frequency, the sizes of the rectangular patches of the unit cell are adjusted such that the unit cell provides the required phase-shift that was obtained in the POS. This procedure can be summarized as follows. First, and for each reflectarray element, two phase shift tables are generated independently for each polarization. Then, the dimensions of the rectangular patches are adjusted using a linear equation. Finally, a zero-finding routine is employed to adjust both dimensions at the same time. During this process, a fixed relative size between the stacked patches is kept, $T_{x_{1}}=0.77T_{x_{2}}$ and $T_{y_{1}}=0.77T_{y_{2}}$. Further details on the GIA applied to POS may be found in [21,33].

Since the employed algorithm is a local optimizer, the starting point of the optimization is very important. Thus, the starting phase-shift distribution will be such that it provides a focused near field at the center of the coverage zone. To that end, the conjugate-phase approach [34] is employed, which establishes that the phase of the field at the aperture should be:

(35) $$∠{\vec{E}}_{\mathrm{ap},i}=k_{0}\;\left|\vec{r}-{\vec{r}}_{i}\;{\;}^{\prime }\right|,$$

for i=1,⋯,N. On the other hand, the phase of the field at the aperture is also equal to the phase introduced by the unit cell plus the phase of the incident field from the feed [32]:

(36) $$∠{\vec{E}}_{\mathrm{ap},i}=-k_{0}d_{i}+∠\rho ,$$

where $d_{i}$ is the distance from the feed phase center to the *i*-th unit cell, and ρ is a direct reflection coefficient (either $\rho_{xx}$ or $\rho_{yy}$, depending on the polarization). Thus, by combining (35) and (36), we can obtain the phase-shift introduced by each reflectarray element to focus the near field at a point $\vec{r}=\left(x,y,z\right)$:

(37) $$∠\rho =k_{0}\left(d_{i}+\left|\vec{r}-{\vec{r}}_{i}\;{\;}^{\prime }\right|\right)=k_{0}\left(d_{i}+\sqrt{{\left(x-x_{i}\right)}^{2}+{\left(y-y_{i}\right)}^{2}+z^{2}}\right).$$

**Figure 8:**
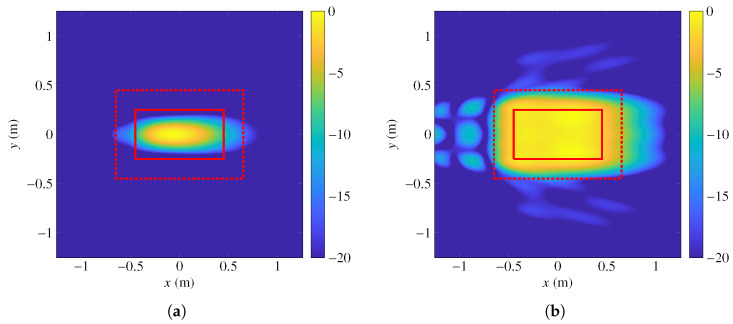
Normalized magnitude of the electric field (**a**) for the starting point of the POS and (**b**) the result of the POS simulated with a MoM-LP at 28 GHz. Coverage zone is represented by a solid red line and transition zone by a dashed red line. Near field is plotted in the NFCS.

Figure 8a shows the focused near field for linear polarization *X* when the phases in (37) are implemented. The magnitude of the field is concentrated in the middle of the coverage zone, but it presents an important drop towards the edges of the coverage zone. Indeed, the ripple in this case is 48 dB for polarization *X* and 38 dB for polarization *Y*. After the POS is completed, a phase-shift distribution is obtained such that the ripple is considerably reduced at 28 GHz. A layout is obtained from this phase-shift distribution by following the steps described above and in [23] and using the MoM-LP described in [35]. When this layout is simulated, the near field shown in Figure 8b is obtained. As can be seen, the near field presents a more uniform distribution in the coverage area when compared to that of the starting point of the POS shown in Figure 8a. Now, the ripple for polarizations X and Y is 2.66 dB and 2.48 dB, respectively. Although it does not comply with the specifications of a maximum ripple of 2 dB, it is a net improvement over the starting point.

However, we are also interested in the performance in the 5G NR n257 band (26.5 –29.5 GHz). To that end, Table 3 gathers the results at seven equispaced frequencies, showing the maximum achieved ripple as well as the percentage of the coverage area that complies with a 1 dB, 2 dB, or 3 dB ripple. As can be seen, the best results are obtained at the frequency of design, 28 GHz. For frequencies 500 MHz above and below 28 GHz, the results deteriorate slightly. However, as the frequency moves further away from the design one, the results quickly worsen, showing the narrow bandwidth behaviour of the reflectarray antenna and the need for a wideband optimization.

### 4.4. Multi-Frequency Optimization of the Near Field

The multi-frequency optimization of the near field consists in a direct layout optimization using the GIA [21]. To accommodate specifications at a number of frequencies, the cost function (*F*) in the backward projector of the GIA is modified in the following fashion [36]:

(38) $$F_{\Omega }=\sum_{f=1}^{N_{f}}\sum_{l=1}^{L}w_{f}\left({\vec{r}}_{l}\right){\left({\left|{\vec{E}}_{\mathrm{NF},f}\left({\vec{r}}_{l}\right)\right|}^{2}-{\left|{\tilde{\vec{E}}}_{\mathrm{NF},f}\left({\vec{r}}_{l}\right)\right|}^{2}\right)}^{2}\Delta \Omega ,$$

where Ω is the region where the near field is computed; $N_{f}$ is the total number of frequencies at which the direct layout optimization is performed; *L* is the number of points in which the region Ω is discretized; $w_{f}$ is a weighting function that depends on the frequency and observation point ${\vec{r}}_{l}$; ${\vec{E}}_{\mathrm{NF},f}\left({\vec{r}}_{l}\right)$ is the computed near field at each iteration of the GIA and frequency; ${\tilde{\vec{E}}}_{\mathrm{NF},f}\left({\vec{r}}_{l}\right)$ is the reference near field in region Ω resulting from applying the specification templates at a given frequency; and ΔΩ is the step of the discretization. Further details on the specifics of the optimization algorithm may be consulted elsewhere [21].

Unlike in POS, where the optimizing variables (sometimes also referred to as degrees of freedom or DoF) were the phases of the direct coefficients, the direct layout optimization employs the geometrical features of the unit cell as optimizing variables. In the present case, the dimensions of the rectangular patches are employed, $T_{x_{1}}$, $T_{x_{2}}$, $T_{y_{1}}$, and $T_{y_{2}}$ (see Figure 6). Thus, a total of 7744 DoF are available for the optimization. Moreover, the direct layout optimization will be carried out in several stages in order to facilitate convergence towards a suitable solution. First, only variables $T_{x_{1}}$, and $T_{x_{2}}$ will be considered at three frequencies, 27 GHz, 28 GHz and 29 GHz. In the second stage, the near field is optimized at the same three frequencies, but this time only considering as variables $T_{y_{1}}$ and $T_{y_{2}}$. Finally, in the third stage, all four variables per element are used in the optimization at the same seven frequencies of Table 3. In all stages, the specifications for both linear polarizations are imposed. In addition, the MoM-LP tool is directly employed in the optimization loop to obtain the real electromagnetic response of the unit cell at each considered frequency. In order to accelerate computations, the differential contributions technique [37] is used.

**Figure 9:**
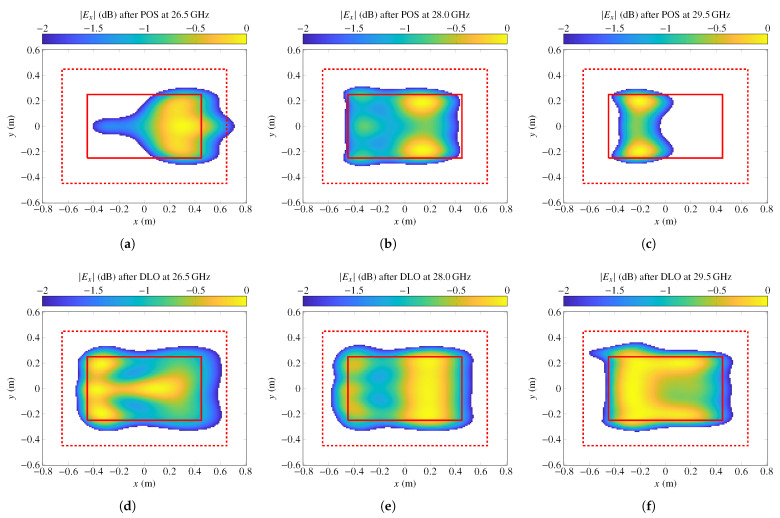
Representation of the normalized electric field magnitude for linear polarization *X* that complies with a ripple of 2 dB at 26.5 GHz (**left** column), 28 GHz (**middle** column), and 29.5 GHz (**right** column) generated by the layout obtained after the POS (**top** row) and after the direct layout optimization (DLO) with the GIA (**bottom** row). Coverage zone is represented by a solid red line and transition zone by a dashed red line. Near field is plotted in the NFCS.

The improvement in the coverage zone can be visually seen in Figure 9, where the normalized magnitude of the electric field for linear polarization *X* is represented at three different frequencies (26.5 GHz, 28 GHz, and 29.5 GHz) before and after the direct layout optimization. The field is only plotted at those points that comply with the requirement of 2 dB of ripple. Although the starting layout does not radiate a near field that fully complies with specifications at 28 GHz, Figure 9b shows that most of the coverage zone does comply, and only at one edge is the ripple higher than 2 dB, demonstrating the successful optimization carried out by the POS. However, due to the intrinsic narrow bandwidth behaviour of the reflectarray, at other frequencies, it does not comply. Nevertheless, after the direct layout optimization, in which the sizes of the two stacked rectangular patches are directly optimized, the antenna is able to provide full coverage in the designated area with a maximum ripple lower than 2 dB.

Table 4 gathers the performance of the optimized layout at seven equispaced frequencies covering the whole 5G NR n257 band. Now, a compliance of 100% is achieved in the whole band for a ripple of 2 dB. Indeed, the worst achieved ripple is 1.5 dB at 29.5 GHz for polarization *X*. When compared with the results shown in Table 3, the worst ripple has been reduced more than 7.5 dB, and even the compliance for a ripple of 1 dB, although not considered a requirement, has improved from the lowest value of 26.4% for polarization *X* at 29.5 GHz to 76.7% for polarization *X* at 28.5 GHz. In the case of the 2 dB compliance columns, the minimum value before the direct layout optimization was 44.3%, which was improved to a 100% compliance after a successful wideband optimization.

### 4.5. Discussion

The proposed wideband near field beam-shaping technique was able to achieve a maximum ripple of 1.5 dB in the whole 5G NR n257 band, corresponding to a 10.7% relative bandwidth. It is worth mentioning that specifications have been met employing a unit cell consisting of two stacked rectangular microstrip patches backed by a ground plane, offering up to four degrees of freedom to perform a direct layout optimization. However, bandwidth could be further improved by employing a more suitable unit cell. For instance, the three stacked patches configuration is known to offer higher bandwidth of operation than only two layers [38]. Alternatively, a unit cell comprised of two layers of parallel and coplanar dipoles is known to be able to offer, after a direct layout optimization, relative bandwidths of up to 20% [36]. When applied to near field beam-shaping, this would allow offering coverage in both the n257 and n258 bands, covering from 24.25 GHz up to 29.5 GHz, which represents 19.5% of relative bandwidth. Additionally, even though the use of a MoM-LP tool directly in the optimization loop penalizes computational performance to achieve wideband results, some techniques could be employed to accelerate the process. In this work, the differential contributions technique [37] was used to accelerate some building blocks of the GIA. However, the unit cell analysis could also be sped up by substituting the MoM-LP with a database of reflection coefficients [39] or the use of machine learning techniques [40].

## 5. Conclusions

This work has presented two near field models for the analysis of spatially fed array antennas, namely, reflectarrays and transmitarrays. The first model is based on the direct application of the radiation equations directly derived from the A and F vector potentials. The volumetric integrals are particularized for a planar array discretized into a number of rectangular unit cells, and thus they are reduced to a summation of a double integral over each unit cell. In turn, this integral is numerically evaluated by a quadrature method, for instance, the mid point quadrature. The second model is based on the superposition of the far field radiated by each array element. The far field is computed using Love’s principle of equivalence assuming a constant field at each unit cell. Both near field models were compared by computing the field radiated by a transmitarray antenna. For the model based on the radiation equations, the near field was also computed for several discretizations of the unit cell for the numerical computation of the double integrals. Both models provide very similar results. When the model based on the radiation equations is used as reference, the relative error in the radiated field obtained with the other model quickly drops as the field is computed further away from the array. With relative errors lower than 3% in the computed tangential near field at 13 λ or further from the array, the model is effectively validated for subsequent use in optimization algorithms for near field beam-shaping.

Then, the model based on far field superposition was applied to perform near field beam-shaping. The considered scenario is aimed at providing indoor coverage in an office for mm-Wave 5G NR networks in the n257 band. The proposed wideband near field optimization strategy is composed of several stages to facilitate convergence towards a suitable solution. First, a phase-only synthesis (POS) was carried out to obtain a reflectarray layout that provides coverage at central frequency (28 GHz). Due to the narrow bandwidth nature of spatially fed planar array antennas, poor compliance is achieved at other frequencies within the n257 band. Thus, a multi-frequency optimization was then carried out employing a method of moments based on local periodicity (MoM-LP) directly in the optimization loop along with the near field model. The cost function of the optimization algorithm was modified to include requirements at several frequencies in the near field region of interest. At the same time, the optimization was carried out in several stages, gradually increasing the number of DoF to improve convergence. Eventually, the optimized antenna complied with the imposed specifications, achieving a magnitude ripple equal or lower than 1.5 dB in the whole 5G NR n257 band, corresponding to a 10.7% relative bandwidth, showing the suitability of the proposed methodology for the wideband near field beam-shaping of spatially fed arrays.

## Figures and Tables

**Table 1:** Relative error of the field components calculated with the different near field models when compared with the model based on the radiation equations with $N_{x}=N_{y}=5$, which is taken as reference.

	z=0.1m			z=1m		
	$E_{x}$	$E_{y}$	$E_{z}$	$E_{x}$	$E_{y}$	$E_{z}$
FF contributions	2.22%	2.21%	3.20%	0.22%	0.22%	0.32%
NF ($N_{x}=N_{y}=1$)	0.09%	0.33%	3.10%	0.06%	0.14%	0.15%
NF ($N_{x}=N_{y}=3$)	0.01%	0.02%	0.18%	0.00%	0.01%	0.01%

**Table 2:** Computational performance of each algorithm in the computation of the near field in a total of 22,500 points employing an Intel i9-9900 CPU working at 3.1 GHz. Computations are parallelized with OpenMP [30].

Model	Time (s)
FF contributions	1.40
NF ($N_{x}=N_{y}=1$)	24.65
NF ($N_{x}=N_{y}=3$)	168.45
NF ($N_{x}=N_{y}=5$)	455.89

**Table 3:** Performance of the layout obtained after a POS at 28 GHz in the whole 5G NR n257 band. X dB (%) indicates what percentage of the coverage area complies with a ripple of X dB.

Frequency (GHz)	Polarization X				Polarization Y			
	Ripple (dB)	1 dB (%)	2 dB (%)	3 dB (%)	Ripple (dB)	1 dB (%)	2 dB (%)	3 dB (%)
26.5	5.34	42.45	63.35	82.03	5.92	30.40	58.80	78.97
27.0	3.79	47.17	71.23	98.79	3.97	43.83	73.20	98.45
27.5	2.53	43.63	93.75	100	2.53	48.57	89.96	100
28.0	2.66	51.28	96.44	100	2.48	45.14	99.10	100
28.5	3.74	58.82	86.04	96.40	3.58	52.70	90.61	98.49
29.0	6.39	33.76	54.02	81.71	5.03	54.69	81.71	89.96
29.5	9.19	26.42	44.32	57.55	7.37	37.15	63.18	77.81

**Table 4:** Performance of the optimized layout in the whole 5G NR n257 band. X dB (%) indicates what percentage of the coverage area complies with a ripple of X dB.

Frequency (GHz)	Polarization X				Polarization Y			
	Ripple (dB)	1 dB (%)	2 dB (%)	3 dB (%)	Ripple (dB)	1 dB (%)	2 dB (%)	3 dB (%)
26.5	1.30	80.89	100	100	0.82	100	100	100
27.0	1.03	99.63	100	100	0.84	100	100	100
27.5	1.08	95.63	100	100	0.96	100	100	100
28.0	1.23	84.90	100	100	0.96	100	100	100
28.5	1.26	76.73	100	100	1.02	99.07	100	100
29.0	1.25	96.72	100	100	1.16	93.67	100	100
29.5	1.50	89.05	100	100	1.49	90.52	100	100

**Figure A1:**
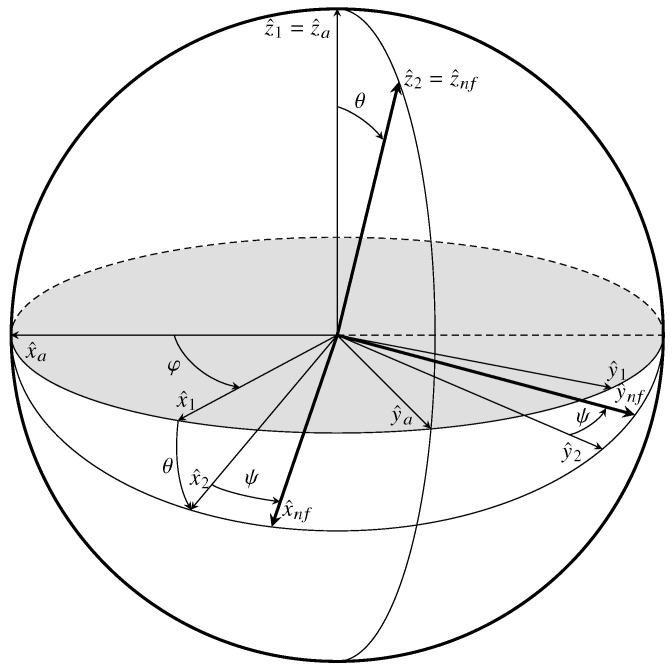
Rotation of the array coordinate system (ACS) $\left({\hat{x}}_{a},{\hat{y}}_{a},{\hat{z}}_{a}\right)$ to obtain the near field coordinate system (NFCS) $\left({\hat{x}}_{n\;f},{\hat{y}}_{n\;f},{\hat{z}}_{n\;f}\right)$. This rotation is specified by angles θ, φ, and ψ.

## Data Availability

Not applicable.

## Abbreviations

The following abbreviations are used in this manuscript:

5G NR	Fifth generation new radio
ACS	Array coordinate system
DLO	Direct layout optimization
DoF	Degrees of freedom
FF	Far field
FFT	Fast Fourier transform
FR	Frequency Range
GIA	Generalized intersection approach
MoM-LP	Method of moments based on local periodicity
NF	Near field
NFCS	Near field coordinate system
POS	Phase-only synthesis

## Appendix A

Computations of the radiated field by the array were carried out in the ACS $\left({\hat{x}}_{a},{\hat{y}}_{a},{\hat{z}}_{a}\right)$. However, the final field must be expressed in the NFCS $\left({\hat{x}}_{n\;f},{\hat{y}}_{n\;f},{\hat{z}}_{n\;f}\right)$. In general, the rotation of the NFCS with regard to the ACS can be expressed by three angles, θ, φ, and ψ, as described in [41]. The definition of θ and φ in spherical coordinates is the usual, while ψ defines a rotation around azis ${\hat{z}}_{2}$, as shown in Figure A1.

The unit vectors of the rotated coordinate system are defined as [41]:

(A1a) $$\begin{array}{cc} {\hat{x}}_{n\;f} & =\hat{\theta }\cos\left(\varphi -\psi \right)-\hat{\varphi }\sin\left(\varphi -\psi \right), \end{array}$$

(A1b) $$\begin{array}{cc} {\hat{y}}_{n\;f} & =\hat{\theta }\sin\left(\varphi -\psi \right)+\hat{\varphi }\cos\left(\varphi -\psi \right), \end{array}$$

(A1c) $$\begin{array}{cc} {\hat{z}}_{n\;f} & =\hat{r}, \end{array}$$

where

(A2a) $$\begin{array}{cc} \hat{\theta } & ={\hat{x}}_{a}\cos\theta \cos\varphi +{\hat{y}}_{a}\cos\theta \sin\varphi -{\hat{z}}_{a}\sin\theta , \end{array}$$

(A2b) $$\begin{array}{cc} \hat{\varphi } & =-{\hat{x}}_{a}\sin\varphi +{\hat{y}}_{a}\cos\varphi , \end{array}$$

(A2c) $$\begin{array}{cc} \hat{r} & ={\hat{x}}_{a}\sin\theta \cos\varphi +{\hat{y}}_{a}\sin\theta \sin\varphi +{\hat{z}}_{a}\cos\theta . \end{array}$$

Substituting (A2) into (A1) yields:

(A3a) $$\begin{array}{cc} {\hat{x}}_{n\;f}= & {\hat{x}}_{a}\left[\cos\theta \cos\varphi \cos\left(\varphi -\psi \right)+\sin\varphi \sin\left(\varphi -\psi \right)\right]+\\ & {\hat{y}}_{a}\left[\cos\theta \sin\varphi \cos\left(\varphi -\psi \right)-\cos\varphi \sin\left(\varphi -\psi \right)\right]-\\ & {\hat{z}}_{a}\sin\theta \cos\left(\varphi -\psi \right), \end{array}$$

(A3b) $$\begin{array}{cc} {\hat{y}}_{n\;f}= & {\hat{x}}_{a}\left[\cos\theta \cos\varphi \sin\left(\varphi -\psi \right)-\sin\varphi \cos\left(\varphi -\psi \right)\right]+\\ & {\hat{y}}_{a}\left[\cos\theta \sin\varphi \cos\left(\varphi -\psi \right)-\cos\varphi \sin\left(\varphi -\psi \right)\right]-\\ & {\hat{z}}_{a}\sin\theta \sin\left(\varphi -\psi \right), \end{array}$$

(A3c) $$\begin{array}{cc} {\hat{z}}_{n\;f}= & {\hat{x}}_{a}\sin\theta \cos\varphi +{\hat{y}}_{a}\sin\theta \sin\varphi +{\hat{z}}_{a}\cos\theta . \end{array}$$

Then, if ${\vec{r}}_{a}={\left(x,y,z\right)}_{\mathrm{ACS}}$ is a point in the ACS, and ${\vec{r}}_{n\;f}={\left(x,y,z\right)}_{\mathrm{NFCS}}$ is the same point in the NFCS, the matrix of change of coordinates from the ACS to the NFCS is:

(A4) $${\vec{r}}_{n\;f}=T\;{\vec{r}}_{a},$$

with:

(A5) $$T=\left(\begin{array}{ccc} {\hat{x}}_{n\;f}\cdot {\hat{x}}_{a} & {\hat{x}}_{n\;f}\cdot {\hat{y}}_{a} & {\hat{x}}_{n\;f}\cdot {\hat{z}}_{a}\\ {\hat{y}}_{n\;f}\cdot {\hat{x}}_{a} & {\hat{y}}_{n\;f}\cdot {\hat{y}}_{a} & {\hat{y}}_{n\;f}\cdot {\hat{z}}_{a}\\ {\hat{z}}_{n\;f}\cdot {\hat{x}}_{a} & {\hat{z}}_{n\;f}\cdot {\hat{y}}_{a} & {\hat{z}}_{n\;f}\cdot {\hat{z}}_{a} \end{array}\right).$$

Conversely, to perform a change of coordinates from the NFCS to the ACS we can calculate:

(A6) $${\vec{r}}_{a}=T^{T}\;{\vec{r}}_{n\;f},$$

where $T^{T}$ is the transpose of *T*, which is an orthogonal matrix, i.e., $T^{T}=T^{-1}$.
